# Direct Comparison of the Lowest Effect Concentrations of Mutagenic Reference Substances in Two Ames Test Formats

**DOI:** 10.3390/toxics9070152

**Published:** 2021-06-29

**Authors:** Bernhard Rainer, Elisabeth Pinter, Lukas Prielinger, Chiara Coppola, Maricel Marin-Kuan, Benoit Schilter, Silvia Apprich, Manfred Tacker

**Affiliations:** 1 Department of Applied Life Sciences, University of Applied Sciences, FH Campus Wien, 1030 Vienna, Austria; elisabeth.pinter@fh-campuswien.ac.at (E.P.); lukas.prielinger@fh-campuswien.ac.at (L.P.); Chiara.Coppola@fh-campuswien.ac.at (C.C.); silvia.apprich@fh-campuswien.ac.at (S.A.); manfred.tacker@fh-campuswien.ac.at (M.T.); 2 Nestle Research-Chemical Food Safety, 1000 Lausanne, Switzerland; maricel.marin-kuan@rdls.nestle.com (M.M.-K.); benoit.schilter@rdls.nestle.com (B.S.)

**Keywords:** complex mixtures, mutagenicity, genotoxicity, Ames assay, food contact materials, bacterial reverse mutation, lowest effective concentration (LEC), S9 comparison

## Abstract

The Ames assay is the standard assay for identifying DNA-reactive genotoxic substances. Multiple formats are available and the correct choice of an assay protocol is essential for achieving optimal performance, including fit for purpose detection limits and required screening capacity. In the present study, a comparison of those parameters between two commonly used formats, the standard pre-incubation Ames test and the liquid-based Ames MPF™, was performed. For that purpose, twenty-one substances with various modes of action were chosen and tested for their lowest effect concentrations (LEC) with both tests. In addition, two sources of rat liver homogenate S9 fraction, Aroclor 1254-induced and phenobarbital/β-naphthoflavone induced, were compared in the Ames MPF™. Overall, the standard pre-incubation Ames and the Ames MPF™ assay showed high concordance (>90%) for mutagenic vs. non-mutagenic compound classification. The LEC values of the Ames MPF™ format were lower for 17 of the 21 of the selected test substances. The S9 source had no impact on the test results. This leads to the conclusion that the liquid-based Ames MPF™ assay format provides screening advantages when low concentrations are relevant, such as in the testing of complex mixtures.

## 1. Introduction

In multiple fields dealing with chemical safety, the Ames test plays an important role for the detection of DNA-reactive genotoxic substances (mutagens) and is recommended to be included as part of a battery of genetic toxicology tests by EFSA [1]. The fields of application also include environmental toxicology, where soil, air or water sample testing is concerned [2,3,4,5]. In addition, the detection of mutagenic impurities in pharmaceutical drugs, as outlined in the ICH M7 guideline [6,7], or in the frame of the development of novel medical products are major topics [8], requires the use of the Ames test. Further applications include food safety assessment [9], safety evaluation of packaging materials [10,11], testing of medical plant extracts [12] or testing materials of importance for the chemical industry such as mineral oils [13]. Overall, those areas raise a common issue, which is the need to assess the mutagenicity of low-level contaminants potentially present in complex mixtures.

The lowest effect concentration (LEC) achieved in the assay, reflecting the limit of detection of mutagens, is the key attribute of the test to address this challenge. Indeed, it has to be low enough to meet regulatory/safety requirements and this in the presence of complex sample matrices, which may interfere with the test results. In this context, the LEC refers to the lowest measured concentration of a mutagenic substance that causes a measurable effect on the test bacteria strains. Together with a (hypothetical) concentration factor that can be achieved during sample preparation, LEC values can be converted into limits of biological detection (LOBD) of the test procedure, which refers to the lowest concentration of a substance that can be detected in a sample [10].

### 1.1. Relevance for Packaging Safety Assessment

Previous investigations [14,15,16] focused on the applicability of in vitro genotoxicity assays for packaging migrate safety assessment. Packaging migrates are typical complex mixtures which could contain low levels of genotoxic chemicals. It was found that the Ames test is currently the most appropriate in vitro bioassay to address the challenges of direct DNA-reactive substances potentially migrating from food contact materials (FCM) into product simulants. Its use has been recommended by an expert group of the International Life Science Institute [10] as part of a comprehensive safety assessment strategy. Compared to other in vitro tests based on mammalian cells, the Ames test exhibits several advantages, such as lower LEC values [14,16] for most substances, possibly resulting from tolerance to higher solvent concentrations [17,18].

### 1.2. Ames Test Protocols and S9 Selection

Different Ames tests formats are available with potential impact on LECs/LOBDs for mutagenic substances [10]. However, the question regarding the most suitable Ames test protocol for detecting very low concentrations of direct DNA-reactive genotoxic contaminants is still open. The initial version of the assay, based on agar media and Petri dishes, is still widely considered the standard format, as it is recommended for regulatory testing and is part of the OECD guideline No 471 [19]. Over the years, many miniaturized formats have emerged [20,21,22,23,24,25]. Most of them still use agar-based media and rely on counting revertant colonies, while new approaches based on respiratory activity measurement [26] are currently being developed. In this context, the Ames MPF™ assay, a liquid incubation format followed by a colorimetric readout, has been promoted as an alternative. This type of liquid incubation assay has been widely applied for testing pharmaceutical substances [7] and herbal formulations [27]. Moreover, recent results showing the feasibility of screening small volumes of FCM migrate samples [15], prompted a detailed look at this version of the assay.

Therefore, the present study compares the LEC values of two Ames formats, namely the pre-incubation standard Petri dish agar Ames test and the Ames MPF™ test. The question of concordance in terms of sensitivity/specificity of these formats was previously addressed [24,28]. However, the performance of these test protocols in terms of achievable LECs/LOBDs has never been directly compared. The LEC refers to the lowest measured concentration of the testing substance able to induce the growth of revertant colonies at equal or higher levels to the threshold established for each bacterial strain according the spontaneous revertant colonies of the solvent control. For this purpose, both test protocols were performed in parallel with 21 chemicals, and the results were compared.

Other than the assay protocol itself, another factor that could theoretically affect the LECs is the metabolic activation system. The production of the most commonly used S9 from Aroclor 1254-induced rat liver homogenate is being phased out, since the production of polychlorinated biphenyls was banned in the late 1970s [29] and stocks are now running out. An efficient and comparable alternative is essential to provide reliable test results in the long term. There are several replacement products on the market, however phenobarbital/β-naphthoflavone (PB/βNF)-induced S9 in particular stands out as a potential promising candidate. To determine the impact of changing the metabolic activation system on the bacterial response and therefore on the LEC values, several Ames-positive test substances were tested with different sources of S9 fractions.

## 2. Materials and Methods

### 2.1. Test Substances, Chemicals and Reagents

Twenty substances classified as mutagenic were analysed for the comparison of the LEC values of the standard pre-incubation Petri dish agar-based Ames and the Ames MPF™. They were mainly selected from the EU Reference Laboratory for Alternatives to Animal Testing (ECVAM) list of recommended chemicals [30]. Another substance, namely melamine, was included as an Ames negative substance and is a known non-genotoxic carcinogen classified as ECVAM category III. Additional substances, which are not part of the ECVAM list, were included to cover other properties, such as higher volatility (formaldehyde) or the interference of coloured substances with the colour shift of the MPF medium (acridine orange). Standard positive control substances (e.g., 2-aminoanthracene or 2-nitrofluorene) were included to allow for an easy comparison of the results with the data of other laboratories, due to general availability. Lastly, two packaging-related substances (phenylglycidyl ether and triglycidyl isocyanurate) as well as a weak positive substance, with a tendency to cause cytotoxic effects and precipitation in higher concentrations during dose-finding experiments (benzo[a]anthracene), were included. All test substances were dissolved and diluted in DMSO. Information about the supplier and purity of the substance is listed in Table 1. Reagents for the Ames MPF™ Assay, namely the exposure and indicator media, were supplied by Xenometrix (Allschwil, Switzerland). For the standard pre-incubation Petri dish agar-based Ames, the protocol by Proudlock [31] was followed and all chemicals were obtained from Carl Roth (Karlsruhe, Germany), except for nutrient broth No 2, which was purchased from Thermo Fisher (Waltham, MA, USA).

### 2.2. Test Strains and Pre-Culture

Two strains were used for the present study: *Salmonella typhimurium* TA98 and TA100, which were supplied by Xenometrix AG. They were grown in an environmental shaker at 250 rpm in Nutrient broth No.2 (Thermo Fisher) with 50 µg/mL ampicillin, until they reached an OD_600_ of 2–2.5 measured with a UV/VIS spectrometer Lambda 265 (Perkin Elmer, Waltham, MA, USA). For the strain TA100, the overnight cultures were pre-screened to test whether the spontaneous background reversion rate was in an acceptable range, according to the Ames MPF™ protocol.

### 2.3. Metabolic Activation

For the comparison test runs, the induced rat liver post-mitochondrial supernatant S9 fractions from phenobarbital/β-naphthoflavone (PB/βNF) and 1254 aroclor (both purchased from Xenometrix) were used. The co-factors were prepared according to Proudlock, 2016 [31]: 5 mM glucose-6-phosphate, 4 mM NADP, 8 mM MgCl_2_, 33 mM KCl in 100 mM sodium phosphate buffer at a pH of ~7.4.

### 2.4. Test Conditions for the Direct Comparison

**Figure 1:**
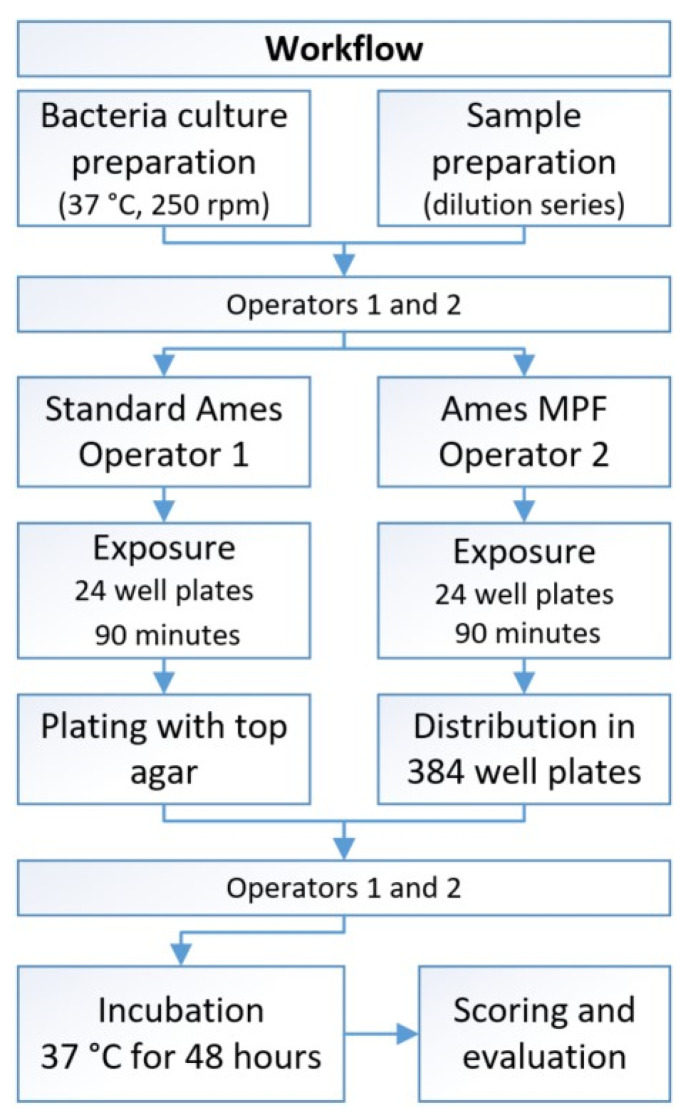
Visualization of the workflow for the comparison of the different Ames protocols.

The following testing workflow (outlined in Figure 1) was chosen to minimise sources of external variation and to ensure a direct comparison is possible: All dilutions were performed in half-logarithmic steps (factor 3.16) and eight concentrations were applied in both assays. The results were scored and documented at the same time point (after 42–54 h). For an assay to be considered valid, the spontaneous revertant background and the positive control response had to be within the confirmed reported range.

### 2.5. Ames MPF™ Test Protocol

The Ames MPF™ test protocol was performed, according to the method’s supplier protocol (Xenometrix), with minor adaptations. As solvent control, DMSO was applied. As a positive control, for TA98 without S9 50 µg/mL 2-nitrofluorene (2NF), for TA100 without S9 2.5 µg/mL 4-nitroquinoline-1-oxide (4NQO) and for TA98 and TA100 with S9 50 µg/mL 2-aminoanthracene (2AA) was applied. Exposures were performed in triplicates in 24-well plates and 10 µL of the test substance or the controls were used per well. The pre-culture was mixed with exposure medium (10% bacteria *v*/*v* for TA98 and 5% *v*/*v* for TA100) and then 240 µL of this mix was added to each well. After 90 min of incubation at 37 °C at 250 rpm in an orbital shaker, 2.6 mL of indicator medium (Xenometrix) were added. The content of the 24-well plates was distributed into three 384-well plates. For the metabolic activation, a 15% S9 mix was prepared and kept on ice, until use and consisted of either PB/β-NF, or Aroclor 1254-induced rat liver S9 and the co-factor mix (see metabolic activation section). The 15% S9 and the co-factor mix were added as required, resulting in a final concentration of 2.25% S9 during the exposure.

### 2.6. Agar-Based Ames Test Protocol

The pre-incubation Petri dish agar-based Ames test protocol was conducted according to the methods described by [31], with minor adaptations. The bacteria were grown as described above and the exposure was done in 24-well plates, in triplicates, containing 100 µL of pre-culture, 500 µL of phosphate buffer (0.2 M, pH 7.4) and 50 µL of the test substance dissolved in DMSO. For the negative control, pure DMSO was applied. As a positive control, for TA98 without S9 50 µg/mL 2NF, for TA100 without S9 2.5 µg/mL 4NQO and for TA98/100 with S9 25 µg/mL 2AA were applied. After 90 min of exposure the mixture was pipetted into 2 mL molten top agar (5 µM histidine and biotin), which was melted and kept at 48 °C in a water bath. The agar was then poured onto Petri dishes containing histidine free minimal glucose agar (MGA; 0.4% glucose). For the metabolic activation, a 1% S9 mix was prepared and kept on ice, until use. It consisted of PB/β-NF-induced rat liver S9 and the co-factor mix (see chapter metabolic activation). The 1% S9 and the co-factor mix were applied instead of the phosphate buffer as required, resulting in a final concentration of 0.77% S9 during the exposure.

### 2.7. Scoring Criteria and Interpretation

The following scoring criteria were applied for both assays: The mean of the solvent control plus one standard deviation was multiplied by a factor of two. This established 2×-factor was set as a positive threshold and test concentrations, for which the mean of revertant/positive wells surpassed this threshold, were considered positive. Toxicity was routinely assessed by checking the background lawn as well as any colour change or bubble formation for the Ames MPF™ protocol.

#### Statistical Analysis

To test whether the mean LECs of the assay formats, or the mean LECs that were ob-tained with two different S9 sources, are significantly different from each other a statistical analysis was conducted. For this purpose a paired sample *t*-test was performed. In order to achieve normal distribution, the LEC values were transformed to their decadic logarithm. Substances for which the assays yielded non-concordant results were excluded.

## 3. Results

### 3.1. Concordance of the Assay Results

The concordance of the positive/negative results was ~90% (19/21 test chemicals). However, two test items yielded discordant results: sodium azide (SA) tested negative in the Ames MPF™, but positive in the standard pre-incubation Petri dish agar-based Ames test (top sample concentration: 25,000 µg/mL, toxicity was observed at higher doses). Incubation with benzo[a]anthrazene (BAA) did not produce a positive test result in the standard pre-incubation Petri dish agar-based Ames test, but tested positive in the Ames MPF™ (top sample concentration: up to 5000 µg/mL, precipitation was observed after adding buffer at the highest dose). The following substances yielded discordant results in only one strain: 2-nitrofluorene (2NF) tested negative in the Ames MPF™ with TA100 − S9, 2-acetylaminofluorene (2AAF) tested negative in the standard pre-incubation Petri dish agar-based Ames test in TA100 + S9 and benzo[a]pyrene (BaP) tested negative in TA100 + S9 in the standard pre-incubation Petri dish agar-based Ames test.

### 3.2. Direct LEC Comparisons

Overall, the 20 standard substances, as well as melamine as negative control, were tested and their LEC values determined in the *Salmonella* strains TA98 and TA100 in both the Ames MPF™ assay and the standard pre-incubation Petri-dish agar-based formats. The mean results of two test runs are listed in Table 2. A more detailed table, which includes the top concentrations for each test run, is provided in the annex (Table A1).

**Figure 2:**
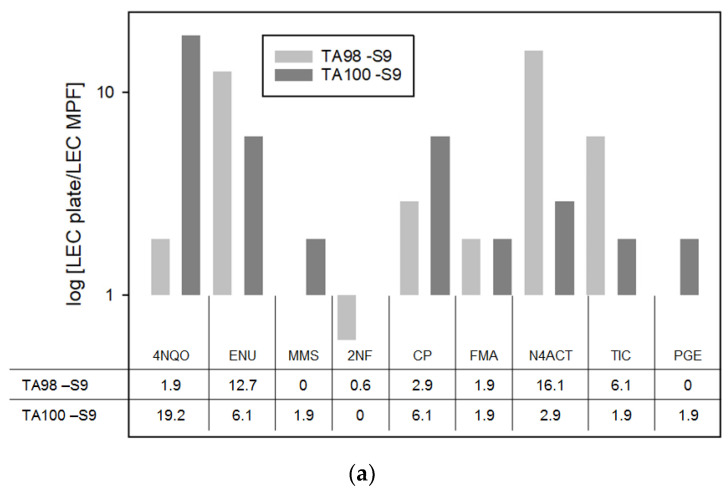

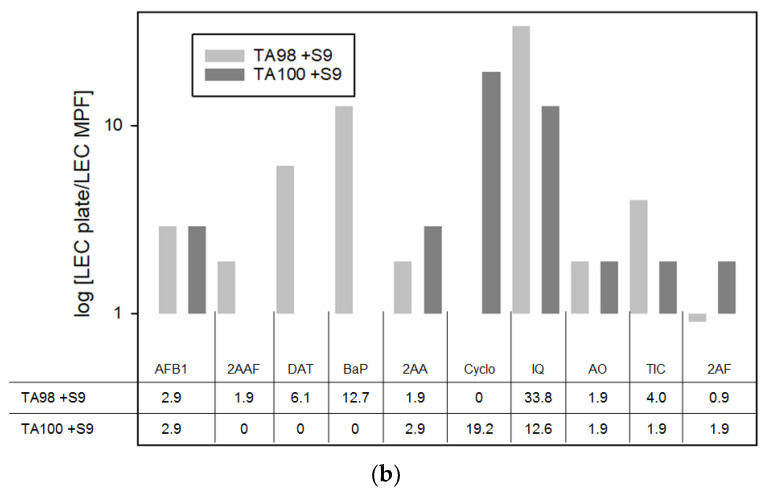
Relative differences in LECs between the Ames MPF and the standard pre-incubation Petri-dish agar-based Ames test are shown in logarithmic scale. The factor was calculated by dividing the mean LEC of the agar-based Ames test by the mean LEC of the Ames MPF. Therefore, a higher factor means a better performance of the Ames MPF assay. (**a**) Test results without metabolic activation, (**b**) results including metabolic activation with PB/ßNP induced S9.

**Figure 3:**
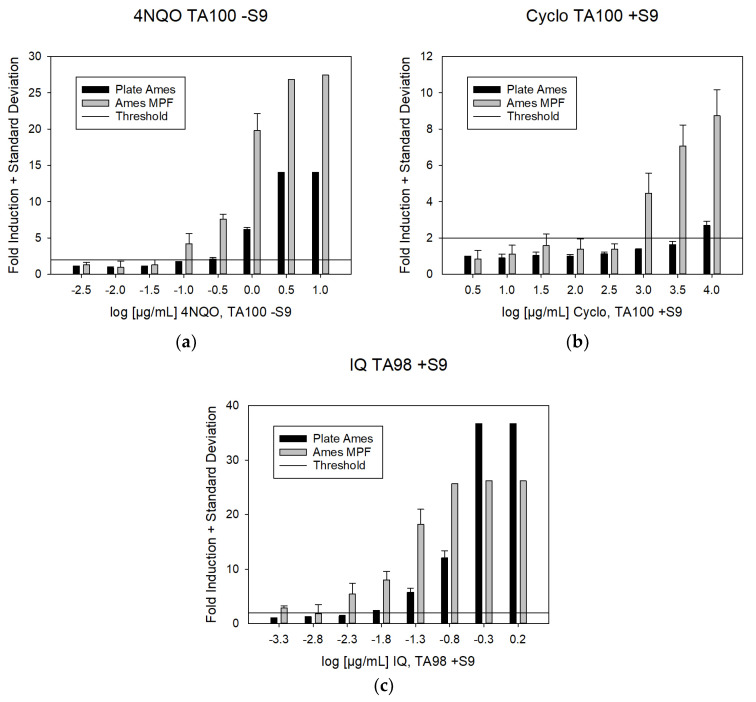
Fold induction of the dose-response testing illustrating differences of dose-responses across test protocols. The bar charts show example results for the dose response curves, obtained with the Ames MPF^TM^ and the standard pre-incubation Petri-dish agar-based Ames assay. The line across the chart indicates the positive threshold, which refers to a two-fold induction over the mean negative control results, including one standard deviation. (**a**) 4NQO tested with TA100, without metabolic activation, (**b**) Cyclo tested with TA100 with metabolic activation and (**c**) IQ tested in TA98 with metabolic activation.

For 81% of the substances (17 out of 21), the arithmetic mean of two independent test runs of the Ames MPF^TM^ yielded lower LEC values, in terms of µg/mL concentration during the incubation, than the standard pre-incubation Petri-dish agar plate Ames test. The mean LEC values for nine out of 11 substances were at least five times lower. Relative differences, for substances which led to positive results in both assay formats, are displayed in Figure 2. Examples of dose response curves, which result in major differences, are shown in Figure 3. The results of both individual test runs can be found in the annex (see annex, Table A1). A statistical analysis of the LEC values for the two assays was conducted including all test runs with concordant results (paired sample *t*-test) and resulted in a highly significant difference (*p* < 0.001). Due to the importance of the LOBD, the overall LECs were also compared in terms of sample concentration (LEC × 25 for the Ames MPF assay and LEC × 13 for the plate Agar assay, see Table A2) and likewise showed highly significant differences (*p* < 0.001). This indicates, that the LEC values obtained with the MPF assay were significantly lower compared to those of the standard pre-incubation petri dish assay and would translate in lower LOBDs.

### 3.3. S9-Source Comparison

**Figure 4:**
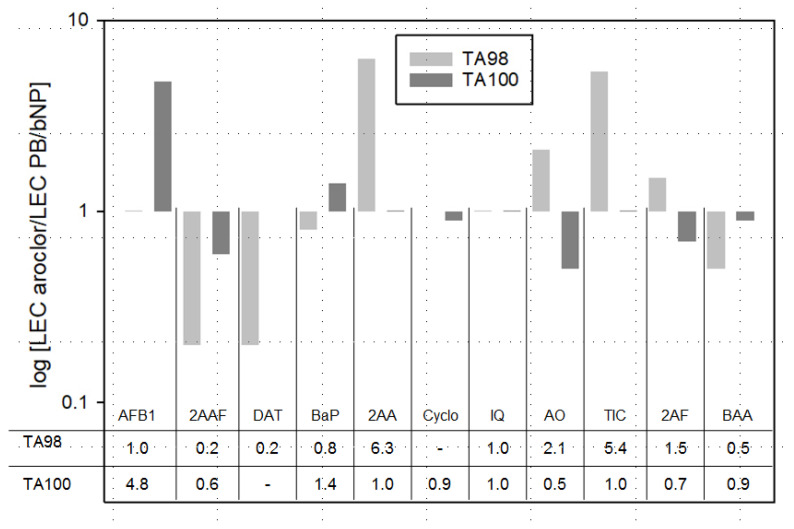
Relative differences in LECs for eleven substances tested with either Aroclor 1254 or PB/β-NP-induced S9, in the Ames MPF™ assay, are shown in logarithmic scale. The factor was calculated by dividing the mean LEC of the Aroclor 1254-induced S9 by the mean LEC of the PB/β-NP-induced S9.

Eleven substances requiring metabolic activation were tested in the Ames MPF™ assay with both Aroclor 1254 and PB/β-NF-induced rat liver S9 fractions. The relative differences in the obtained LECs are shown in Figure 4. All substances tested positive with both Aroclor 1254 and PB/β-NF-induced S9. As expected, slightly different LEC values were obtained for individual substances in the two groups, however statistical analysis revealed no significant overall difference (*p* = 0.65). More detailed information on the individual test runs and exact LECs are shown in the Appendix A (Table A3).

## 4. Discussion

### 4.1. Assay Concordance

While previous publications addressed the issue of assay concordance with a wider range of test substances, the findings in our study, an overall concordance of 90%, align well with previous results [24,28]. Specifically, only 2/21 substances yielded discordant results, namely sodium azide (SA) and benzo[a]anthracene (BAA). The discordance of the results for SA, might be due to a higher bioavailability in the liquid media. The substance is not only mutagenic in bacteria, but also commonly used as an antimicrobial agent, which works by binding to heme-iron (e.g., cytochrome oxidase). However, it is recommended by the OECD 471 guideline as a positive control substance for the strain TA100 [40]. BAA on the other hand showed a tendency during initial experiments, to cause only very weak positive results and increased toxicity, while precipitating in the highest concentrations. More narrow dilution steps, as well as an increase in S9-concentration might have allowed for the detection of this substance in the standard pre-incubation Petri-dish agar-based Ames assay.

### 4.2. LEC According to the Test Protocol

When comparing the LEC values for 20 mutagenic substances, it appears that most of them are detected at a lower concentration with the Ames MPF™ protocol. The mean LECs range from 0.6-fold (2NF) to 33.8-fold (IQ) lower in the Ames MPF™ protocol as compared to values obtained with the standard pre-incubation Petri-dish agar-based Ames protocol. For 81% of the substances (17 out of 21), the Ames MPF™ protocol yielded lower LEC values and for 43% (9 out of 21) of the substances, the difference was at least 5-fold. Overall, these differences are statistically significant with a *p*-value < 0.001. A possible explanations for the lower LECs of the Ames MPF™ protocol could be (according to a publication by Xenometrix, [28]) either potential adsorption effects of the agar therefore reducing bacteria exposure in the standard version and/or an uneven distribution of the test chemicals during the incubation.

There is a significant difference in S9 mix concentration for the Ames MPF™ (2.25%) and the standard pre-incubation Petri-dish agar-based Ames exposure (0.77%). However, in preliminary experiments (data not shown) it was found that lower S9 concentrations yielded slightly better LECs for the agar-based version. Further, studies by Belser et al. [41] and Zeiger et al. [42] showed that less S9 led to an improved detection of BaP and 2AA at lower concentrations. However, they concluded that some substances present at higher concentrations were not as easily detected with less S9. In the study, the aim was to detect at low concentrations to obtain the lowest LECs, therefore it was concluded that the application of less S9 is more suitable.

### 4.3. S9 Fraction Comparison

The results of the S9 comparison (see Figure 4) indicate that both Aroclor 1254-induced, and PB/β-NF-induced rat liver S9 worked equally well. An older study that compared these S9-types, as well as the respective Cyp enzyme activities, came to a similar conclusion [43]. Overall, the differences that can be seen in the substance per substance response in Figure 4 can most likely be explained by varying Cyp activities. While the mean LEC values detected varied from 0.2-fold (2AF) up to 6.3-fold (2AA), the overall differences in the results were inconsistent and not statistically significant in term of overall LEC values (*p* = 0.65). It has to be mentioned, that for the purpose of this study only two batches of S9 were compared and both were subjected to quality control by the supplier. Previous studies have found that significant variations between different types of S9 products are possible [44,45]. However, the present dataset indicates that Aroclor 1254-induced S9, which will not be available anymore in the near future, can likely be adequately replaced by PB/β-NF induced S9, without any anticipated negative impact on the LEC values. In the long term an animal-free S9 source may become preferable [46]. While initial results looks highly promising [44], more data needs to be provided, before such material can be considered a valid alternative.

### 4.4. Implication for the LOBD

As already mentioned, the LOBD for the Ames test, refers to the substance concentration that can be detected in the sample. When comparing the sample concentrations instead of the concentration during the exposure step (in the incubation medium), the differences decrease, but the Ames MPF™ still yields significantly lower results (see Table A3). This type of comparison is however only relevant, when the sample quantities applied during the exposure (4%-Ames MPF and ~8%-standard pre-incubation Petri-dish agar-based Ames test) remain constant. The applicable sample concentration can vary widely, depending on the solvents and can reach up to 70% or more for protocols for water testing, such as in the Ames Aqua [47].

### 4.5. Practical Considerations

When it comes to the practical applicability, the Ames MPF™ protocol offers major advantages compared to the standard pre-incubation Petri dish agar-based Ames test: (i) The amount of sample material required is considerably lower (10 µL per data point for the Ames MPF™ protocol vs. at least 50 µL per data point for the standard pre-incubation Petri dish agar-based Ames test protocol). (ii) Lower amounts of S9 and other consumables are required. (iii) The handling time is much shorter and a single operator can handle at least twice as many samples in the same time.

For the detection of toxic effects of test substances or sample materials, the standard approach is to assess the growth of the bacterial background lawn [31]. While this is not possible for the Ames MPF™ version, colour changes and bubble formation can be an indicator for toxicity [28]. When sample toxicity is a concern, which is the case when testing FCM migrate samples, it has been suggested to use a spiking approach with a well characterized mutagen, a procedure easily applicable to the Ames MPF™ protocol [10,15]. It has to be acknowledged that the procedure can be applied in the standard pre-incubation Petri-dish agar-based Ames test as well, but would lead to an increased requirement in sample volume and material which often is not feasible with complex mixtures and especially for packaging migrants. The spiking approach could also detect other sources of inhibition than cytotoxicity in complex mixtures, since inhibitory effects that are based on other effects (e.g., adsorption on matrix particles) could also be detected.

A disadvantage for the Ames MPF™ assay is the limited number of wells scored, precisely 48 per data point. This results in a non-linear response when the revertant count increases, since multiple events (mutations) can occur in a single well. Therefore, a slight increase in background reversion rate, mostly with TA100, can have an impact on the assay performance, which also negatively affects the LEC and the LOBD. This makes pre-screening of the bacteria pre-cultures for low spontaneous reversion rates a useful tool, in particular when reproducible and low LEC values are of importance.

### 4.6. Relevance for FCM Safety Assessment

According to the threshold of toxicological concern concept (TTC) [48], the suggested acceptable limit of direct DNA reactive mutagenic substances is 0.15 µg/L in the migrate sample. This limit is very conservative and poses a challenge, for both chemical analytical methods and bio-detection approaches [10]. When comparing this to the LEC results, as shown in Table 2, it can be seen that only the two most potent substances, namely IQ and AFB1, could be picked up at such low levels. Specifically, these are highly potent mutagens, which cannot be expected to occur in complex mixtures, such as packaging samples under realistic conditions. However, in a previous publication it was demonstrated that the Ames MPF™ assay is capable to detect mutagenic activity under realistic conditions in FCM migrate samples [15]. Alternative approaches have been proposed combining chemical and bioassays solutions as high performance thin-layer chromatography (HPTLC). Most probably a breakthrough improvement of LOBDs would require a very different test system design such as the coupling of bioassay with high performance thin-layer chromatography [49,50,51]. Finally, when considering the fact that the Ames MPF™ has not only practical advantages, but also provides significantly lower LEC values and LOBDs, its use might be preferred over the standard pre-incubation Petri-dish agar-based Ames test. However, the Ames MPF™ is still not the ideal solution, since further improvements must be made to allow for a more consistent and sensitive detection of low levels of mutagenic contamination, in order to fulfil regulatory requirements.

## 5. Conclusions

- According to the conditions and data analysis applied, the LEC values of the Ames MPF™ assay are significantly lower when compared to the LEC values obtained with the standard pre-incubation Petri-dish agar-based Ames protocol. This is expected to result in lower LOBDs for mutagens in complex mixtures.
- In addition to LEC values, the choice of assay protocol should be based on regulatory requirements as well as technical considerations such as availability of sample material and consumables required.
- The use of either Aroclor 1254-induced S9 or PB/β-NF-induced S9 has no major impact on LEC values.
- The assay protocols show a concordance of over 90% for the set of test chemicals that were chosen for this study.
- Safety assessment of packaging migrate material: Neither protocol can consistently detect DNA reactive substances at a concentration range of 0.15 µg/kg, a limit which is derived from the TTC concept for substances with alert for mutagenicity. More research is needed to achieve such low a level of detection.

Based on the present comparison study, it can be concluded that the Ames MPF™ assay is a suitable approach for screening samples for low concentrations of genotoxic substances. This is of importance, when assessing complex mixtures, such as packaging samples, for low-level contaminations.

## Figures and Tables

**Table 1:** List of test substances used for the direct LEC comparison as well as the S9 comparison.

Chemical	Abbreviation	CAS No.	Purity [%]	Supplier	Selection Criteria/Mode of Action
2,4-Diaminotoluene	DAT	95-80-7	99.5	SCB ^1^	Aromatic amine, requires metabolic activation [29]
2-Acetylaminofluorene	2AAF	53-96-3	≥98	Sigma Aldrich	Hydroxylated by CYP1A2and then acetylated. Forms C^8^ adduct on guanine [29]
2-Amino-3-methylimidazol[4,5-f]quinoline	IQ	76180-96-6	98	SCB ^1^	Heterocyclic amine with potent genotoxicity, requires metabolic activation [29]
4-Nitroquinoline 1-oxide	4NQO	56-57-5	≥98	Sigma Aldrich	Alkylating agent, forms DNA adducts [29]
Aflatoxin B1	AfB1	1162-65-8	≥98	Fermentek	Activated by CYP3A4. Forms various adducts [29]
Benzo[a]pyrene	BaP	50-32-8	≥96	Sigma Aldrich	Requires metabolic activation (CYP 1A1, 1B1, epoxide hydrolase), forms bulky adduct [29]
Cisplatin	CP	15663-27-1	n.s.	Sigma Aldrich	Cross-linking agent [29]
Cyclophosphamide monohydrate	Cyclo	6055-19-2	≥97	SCB ^1^	Requires metabolic activation (CYP2B6) [29]
Melamine	Mel	108-78-1	99	Sigma Aldrich	Ames negative, causes bladder and ureteral carcinomas [29]
Methyl methanesulfonate	MMS	66-27-3	99	Sigma Aldrich	Strong clastogen (N^7^ alkylation) [29]
*N*-ethyl-*N*-nitrosourea	NEU	759-73-9	56	SCB ^1^	Strong gene mutagen (O^6^ alkylation) [29]
2-Aminoanthracene	2AA	613-13-8	96	Carl Roth	Positive control, activated mainly by CYP1A2, DNA binding [32]
2-Aminofluorene	2AF	153-78-6	98	Sigma Aldrich	Positive control, formation of C^8^-AF adducts [33]
2-Nitrofluorene	2NF	607-57-8	>99	TCI ^2^	Positive control, adduct formation [34]
N4-Aminocytidine	N4ACT	57294-74-3	≥95	SCB ^1^	Positive control, DNA incorporation, AT to GC transition [35]
Sodium azide	SA	26628-22-8	≥99.5	Sigma Aldrich	Positive control, A.T to G.C base pair transition and transversion [36]
Formaldehyde	FM	50-00-0	37	SCB ^1^	Volatile, N-hydroxymethyl mono-adducts on guanine, adenine and cytosine, N-methylene crosslinks [37]
Acridine Orange	AO	494-38-2	n.s.	SCB ^1^	Strong coloring agent, DNA intercalation [38]
Benzo[a]anthracene	BAA	56-55-3	99	Sigma Aldrich	S9 Weak positive, adduct formation, oxidative DNA damage [39]
Phenylglycidyl ether	PGE	204-557-2	99	Sigma Aldrich	Packaging related [14]
Triglycidyl isocyanurate	TIC	2451-62-9	≥98	SCB ^1^	Packaging related [14]

^1^ SCB: Santa Cruz Biotechnology, ^2^ TCI: Tokyo Chemical Industry, n.s.: not specified by the supplier.

**Table 2:** Comparison of the Ames MPF™ protocol with the standard pre-incubation Petri-dish agar-based Ames test. Each substance was tested twice with the same dilution series and pre-culture. The concentration in µg/mL refers to the substance concentration during the exposure step. Each substance was tested in eight concentrations in half logarithmic dilution steps. (**a**) test results without metabolic activation, (**b**) results including metabolic activation with PB/ßNP induced S9.

**(a)**					
**Substance**	**CAS**	**Strain TA98 − S9**		**Strain TA100 − S9**	
		**[µg/mL]**		**[µg/mL]**	
		**Plate**	**MPF**	**Plate**	**MPF**
4NQO	56-57-5	0.08	0.04	0.08	0.004
ENU	759-73-9	320	25	154	25
MMS	66-27-3	–	–	77	40
2NF	607-57-8	0.38	1	12	–
CP	15663-27-1	12	4	8	1
FMA	50-00-0	8	4	12	6
SA	26628-22-8	–	–	1.2	–
N4ACT	57294-74-3	67	4	0.012	0.0042
Mel	108-78-1	–	–	–	–
TIC	2451-62-9	127	21	192	100
PGE	204-557-2	–	–	12	6
**(b)**					
**Substance**	**CAS**	**Strain TA98 + S9**		**Strain TA100 + S9**	
		**[µg/mL]**		**[µg/mL]**	
		**Plate**	**MPF**	**Plate**	**MPF**
AFB1	1162-65-8	0.0025	0.0008	0.0077	0.0026
2AAF	53-96-3	0.38	0.2	–	2
DAT	95-80-7	160	26	–	–
BaP	50-32-8	3	0.2	–	0.64
2AA	613-13-8	0.02	0.01	0.2	0.1
Cyclo	6055-19-2	–	–	689	36
IQ	76180-96-6	0.001	0.00002	0.08	0.006
AO	494-38-2	0.19	0.1	1.92	1
Mel	108-78-1	–	–	–	–
TIC	2451-62-9	127	31.6	192	100
2AF	153-78-6	0.038	0	0.8	0.4
BAA	56-55-3	–	35	–	2

## Data Availability

The data presented in this study are available on request from the corresponding author. The data are not publicly available due to privacy restrictions.

## Appendix A

**Table A1:** Comparison of individual results of two independent test runs in the Ames MPF™ protocol with the standard agar-based Ames test. The concentration in µg/mL represents the concentration during the exposure step. The top dose is presented as µg/mL in the sample. Each substance was tested in eight concentrations in half logarithmic dilution steps.

**Strain TA98-Tests Run without Metabolic Activation (−S9)**								
**Substance**	**CAS**	**Run 1 [µg/mL]**		**Top Dose**	**Run 2 [µg/mL]**		**Top Dose**	**Factor**
		**Plate**	**MPF**	**[µg/mL]**	**Plate**	**MPF**	**[µg/mL]**	
4NQO	56-57-5	0.08	0.04	10	0.08	0.04	10	1.9
ENU	759-73-9	487	25	20,000	154	25	20,000	12.7
MMS	66-27-3	–	–	10,000	–	–	10,000	–
2NF	607-57-8	0.4	0.6	5000	0.4	0.6	500	0.6
CP	15663-27-1	12	2	5000	12	6	500	2.9
FMA	50-00-0	12	6	5000	4	2	5000	1.9
SA	26628-22-8	–	–	5000	–	–	25,000	–
N4ACT	57294-74-3	12	6	5000	122	2	5000	16.1
Mel	108-78-1	–	–	25,000	–	–	25,000	–
TIC	2451-62-9	192	10	25,000	61	32	7906	6.1
PGE	204-557-2	–	–	5000	–	–	5000	–
**Strain TA100-Tests Run without Metabolic Activation (−S9)**								
**Substance**	**CAS**	**Run 1 [µg/mL]**		**Top Dose**	**Run 2 [µg/mL]**		**Top Dose**	**Factor**
		**Plate**	**MPF**	**[µg/mL]**	**Plate**	**MPF**	**[µg/mL]**	
4NQO	56-57-5	0.08	0.004	10	0.077	0.004	10	19.2
ENU	759-73-9	154	25	20,000	154	25	20,000	6.1
MMS	66-27-3	77	40	10,000	77	40	10,000	1.9
2NF	607-57-8	12	–	5000	12	–	500	–
CP	15663-27-1	3.8	0.6	5000	12	2	500	6.1
FMA	50-00-0	12	6	5000	12	6	5000	1.9
SA	26628-22-8	0.38	–	5000	1.9	–	25,000	–
N4ACT	57294-74-3	0.012	0.006	5	0.012	0.002	5	2.9
Mel	108-78-1	–	–	25,000	–	–	25,000	–
TIC	2451-62-9	192	100	25,000	192	100	25,000	1.9
PGE	204-557-2	12	6	5000	12	6	5000	1.9
**Strain TA98-Tests Run with Metabolic Activation (+S9)**								
**Substance**	**CAS**	**Run 1 [µg/mL]**		**Top Dose**	**Run 2 [µg/mL]**		**Top Dose**	**Factor**
		**Plate**	**MPF**	**[µg/mL]**	**Plate**	**MPF**	**[µg/mL]**	
AFB1	1162-65-8	0.0025	0.0004	10	0.0025	0.0013	1	2.9
2AAF	53-96-3	0.38	0.2	500	0.38	0.20	500	1.9
DAT	95-80-7	77	40	10,000	243	13	10,000	6.1
BaP	50-32-8	1.2	0.2	5000	3.8	0.2	500	12.7
2AA	613-13-8	0.024	0.013	10	0.024	0.013	10	1.9
Cyclo	6055-19-2	–	–	10,000	–	–	10,000	–
IQ	76180-96-6	0.0012	0.000019	15	0.00012	0.000019	0.15	33.8
AO	494-38-2	0.19	0.10	2500	0.19	0.10	250	1.9
Mel	108-78-1	–	–	25,000	–	–	7906	–
TIC	2451-62-9	61	32	25,000	192	32	7906	4.0
2AF	153-78-6	0.038	0.020	50	0.038	0.063	50	0.9
BAA	56-55-3	–	6	5000	–	63	5000	–
**Strain TA100-Tests Run with Metabolic Activation (+S9)**								
**Substance**	**CAS**	**Run 1 [µg/mL]**		**Top Dose**	**Run 2 [µg/mL]**		**Top Dose**	**Factor**
		**Plate**	**MPF**	**[µg/mL]**	**Plate**	**MPF**	**[µg/mL]**	
AFB1	1162-65-8	0.008	0.004	1	0.0077	0.0013	1	2.9
2AAF	53-96-3	–	2	5000	–	2	5000	–
DAT	95-80-7	–	–	10,000	–	–	10,000	
BaP	50-32-8	–	0.64	5000	–	0.64	500	–
2AA	613-13-8	0.24	0.13	10	0.24	0.04	10	2.9
Cyclo	6055-19-2	769	40	10,000	608	32	25,000	19.2
IQ	76180-96-6	0.036	0.006	15	0.115	0.006	15	12.6
AO	494-38-2	1.9	1.0	250	1.9	1.0	250	1.9
Mel	108-78-1	–	–	25,000	–	–	7906	–
TIC	2451-62-9	192	100	25,000	192	100	25,000	1.9
2AF	153-78-6	0.385	0.632	50	1.2	0.2	50	1.9
BAA	56-55-3	–	2	5000	–	2	5000	–

**Table A2:** Comparison of individual results of two independent test runs in the Ames MPF™ protocol with the standard agar-based Ames test. The concentration in µg/mL represents the substance in the sample. Each substance was tested in eight concentrations in half logarithmic dilution steps.

**Strain TA98-Tests Run without Metabolic Activation (−S9)**								
**Substance**	**CAS**	**Run 1 [µg/mL]**		**Top Dose**	**Run 2 [µg/mL]**		**Top Dose**	**Factor**
		**Plate**	**MPF**	**[µg/mL]**	**Plate**	**MPF**	**[µg/mL]**	
4NQO	56-57-5	1	1	10	1	1	10	1.0
ENU	759-73-9	6325	632.5	20,000	2000	632.5	20,000	6.6
MMS	66-27-3	–	–	10,000	–	–	10,000	–
2NF	607-57-8	5	15.8	5000	5	15.8	500	0.3
CP	15663-27-1	158.1	50	5000	158.1	158.1	500	1.5
FMA	50-00-0	158.1	158.1	5000	50	50	5000	1.0
SA	26628-22-8	–	–	5000	–	–	25,000	–
N4ACT	57294-74-3	158.1	158.1	5000	1581.1	50	5000	8.4
Mel	108-78-1	–	–	25,000	–	–	25,000	–
TIC	2451-62-9	2500	250	25,000	791	791	7906	3.2
PGE	204-557-2	–	–	5000	–	–	5000	–
**Strain TA100-Tests Run without Metabolic Activation (−S9)**								
**Substance**	**CAS**	**Run 1 [µg/mL]**		**Top Dose**	**Run 2 [µg/mL]**		**Top Dose**	**Factor**
		**Plate**	**MPF**	**[µg/mL]**	**Plate**	**MPF**	**[µg/mL]**	
4NQO	56-57-5	1	0.1	10	1	0.1	10	10.0
ENU	759-73-9	2000	632.5	20,000	2000	632.5	20,000	3.2
MMS	66-27-3	1000	1000	10,000	1000	1000	10,000	1.0
2NF	607-57-8	158.1	–	5000	158.1	–	500	–
CP	15663-27-1	50	15.8	5000	158.1	50	500	3.2
FMA	50-00-0	158.1	158.1	5000	158.1	158.1	5000	1.0
SA	26628-22-8	5	–	5000	25	–	25,000	–
N4ACT	57294-74-3	0.158	0.158	5	0.158	0.05	5	1.5
Mel	108-78-1	–	–	25,000	–	–	25,000	–
TIC	2451-62-9	2500	2500	25,000	2500	2500	25,000	1.0
PGE	204-557-2	158.1	158.1	5000	158.1	158.1	5000	1.0
**Strain TA98-Tests Run with Metabolic Activation (+S9)**								
**Substance**	**CAS**	**Run 1 [µg/mL]**		**Top Dose**	**Run 2 [µg/mL]**		**Top Dose**	**Factor**
		**Plate**	**MPF**	**[µg/mL]**	**Plate**	**MPF**	**[µg/mL]**	
AFB1	1162-65-8	0.032	0.01	10	0.032	0.032	1	1.5
2AAF	53-96-3	5	5	500	5	5	500	1.0
DAT	95-80-7	1000	1000	10,000	3162	316	10,000	3.2
BaP	50-32-8	15.8	5	5000	50	5	500	6.6
2AA	613-13-8	0.316	0.316	10	0.316	0.316	10	1.0
Cyclo	6055-19-2	–	–	10,000	–	–	10,000	–
IQ	76180-96-6	0.015	0.00047	15	0.0015	0.00047	0.15	17.6
AO	494-38-2	2.5	2.5	2500	2.5	2.5	250	1.0
Mel	108-78-1	–	–	25,000	–	–	7906	–
TIC	2451-62-9	791	791	25,000	2500	791	7906	2.1
2AF	153-78-6	0.5	0.5	50	0.5	1.581	50	0.5
BAA	56-55-3	–	158.1	5000	–	1581.1	5000	–
**Strain TA100-Tests Run with Metabolic Activation (+S9)**								
**Substance**	**CAS**	**Run 1 [µg/mL]**		**Top Dose**	**Run 2 [µg/mL]**		**Top Dose**	**Factor**
		**Plate**	**MPF**	**[µg/mL]**	**Plate**	**MPF**	**[µg/mL]**	
AFB1	1162-65-8	0.1	0.1	1	0.1	0.032	1	1.5
2AAF	53-96-3	–	50	5000	–	50	5000	–
DAT	95-80-7	–	–	10,000	–	–	10,000	–
BaP	50-32-8	–	16	5000	–	16	500	–
2AA	613-13-8	3.162	3.162	10	3.162	1	10	1.5
Cyclo	6055-19-2	10,000	1000	10,000	7906	791	25,000	10.0
IQ	76180-96-6	0.47	0.15	15	1.5	0.15	15	6.6
AO	494-38-2	25	25	250	25	25	250	1.0
Mel	108-78-1	–	–	25,000	–	–	7906	–
TIC	2451-62-9	2500	2500	25,000	2500	2500	25,000	1.0
2AF	153-78-6	5	15.811	50	15.811	5	50	1.0
BAA	56-55-3	–	50	5000	–	50	5000	–

**Table A3:** Individual results of two independent test runs in the Ames MPF assay with PB/ßNP-induced S9 vs. Aroclor 1254-induced S9. LECs are presented in µg/mL in the exposure medium. The factor is calculated by dividing the mean LEC of the results obtained with Aroclor 1254 by the results obtained with PB/β-NP-induced S9.

**TA98**						
**Substance**	**CAS**	**Arclor 1254 LEC [µg/mL]**		**PB/ßNF LEC [µg/mL]**		**Factor**
		**Run 1**	**Run 2**	**Run 1**	**Run 2**	
AFB1	1162-65-8	0.0013	0.0004	0.0004	0.0013	1.0
2AAF	53-96-3	0.06	0.03	0.20	0.20	0.2
DAT	95-80-7	8	25	126	13	0.2
BaP	50-32-8	0.20	0.12	0.20	0.20	0.8
2AA	613-13-8	0.12	0.04	0.013	0.013	6.3
IQ	76180-96-6	0.000019	0.000019	0.000019	0.000019	1.0
AO	494-38-2	0.1	0.3	0.1	0.1	2.1
TIC	2451-62-9	316	25	32	32	5.4
2AF	153-78-6	0.06	0.06	0.02	0.06	1.5
BAA	56-55-3	20	13	6	63	0.5
**TA 100**						
**Substance**	**CAS**	**Arclor 1254 LEC [µg/mL]**		**PB/ßNF LEC [µg/mL]**		**Factor**
		**Run 1**	**Run 2**	**Run 1**	**Run 2**	
AFB1	1162-65-8	0.013	0.013	0.004	0.001	4.8
2AAF	53-96-3	0.25	2.00	2	2	0.6
BaP	50-32-8	1.0	0.2	0.6	0.6	0.9
2AA	613-13-8	0.13	0.31	0.13	0.04	2.6
Cyclo	6055-19-2	32	100	40	32	1.8
IQ	76180-96-6	0.006	0.006	0.006	0.006	1.0
AO	494-38-2	0.32	0.32	1	1	0.3
TIC	2451-62-9	100	100	100	100	1.0
2AF	153-78-6	0.2	0.6	0.6	0.2	1.0
BAA	56-55-3	1.6	1.6	2	2	0.8
